# Epidemiology, Types, Causes, Clinical Presentation, Diagnosis, and Treatment of Hypothyroidism

**DOI:** 10.7759/cureus.46241

**Published:** 2023-09-30

**Authors:** Udit M Zamwar, Komal N Muneshwar

**Affiliations:** 1 Community Medicine, Jawaharlal Nehru Medical College, Datta Meghe Institute of Higher Education and Research, Wardha, IND

**Keywords:** levothyroxine, deficiency, tsh, thyroxine, hypothyroidism

## Abstract

Hypothyroidism means an underactive thyroid gland. This leads to a decrease in the functioning of the thyroid gland. It is a very common endocrine disorder that causes under-secretion of thyroid hormones, mainly thyroxine (T4) and triiodothyronine (T3). It affects people of every age group but is more commonly found in women and older people. The symptoms of hypothyroidism can go unnoticed, may not be specific, and may overlap with other conditions, which makes it harder to diagnose it in some cases. Common symptoms include fatigue, weight gain, increased sensitivity to cold (cold intolerance), irregular bowel movements (constipation), and dry skin (xeroderma). These conditions are mostly the result of a low metabolic rate in the body. Weight gain occurs due to a decrease in fat-burning rate and cold intolerance due to a decrease in heat production by the body. This condition can be caused by a variety of factors, including autoimmune diseases, radiation therapy, thyroid gland removal surgeries, and certain medications. The diagnosis of hypothyroidism is based on laboratory tests that measure the levels of thyroid hormones (T3 and T4) in the blood. Treatment typically involves lifelong hormone replacement therapy with synthetic thyroid hormone replacement medication, such as levothyroxine, to help regulate hormone levels in the body. People with hypothyroidism may need to have their medication dosage adjusted over time. If hypothyroidism is left untreated, it can lead to severe complications like mental retardation, delayed milestones, etc., in infants and heart failure, infertility, myxedema coma, etc., in adults. With appropriate treatment, the symptoms of hypothyroidism can be effectively managed, and most people with the condition can lead normal, healthy lives. Lifestyle modifications like eating healthy food and exercising regularly can help manage the symptoms and improve the quality of life.

## Introduction and background

Hypothyroidism is a very common pathological condition that is caused by a deficiency of the thyroxine hormone. The thyroxine hormone regulates the metabolism of the body, and its deficiency can cause complications in various organs of the body. It may cause severe effects on the health of the body and ultimately lead to death if it is not treated [1]. Various cases of hypothyroidism can be found in the general population, and these cases lack symptom specificity. Hence, hypothyroidism is defined as being mainly biochemical in nature. Primary hypothyroidism presents with increased levels of thyroid-stimulating hormone (TSH) in the blood, i.e., higher than the normal range, and also with the presence of free thyroxine (T4) concentrations in the blood, which are lower than the normal range. Subclinical hypothyroidism is a mild form of hypothyroidism. Patients with subclinical hypothyroidism present with TSH concentrations that are over the normal range (5-10 mIU/L) and free T4 concentrations that are in the normal reference range [1]. It is still not clear whether to use the current normal reference ranges of TSH and T4 to test for impaired thyroid function. This problem is clinically very important, as the tests used to check for hypothyroidism are based on these reference values and serve as the basis for the treatment of hypothyroidism. Replacement of T4 with levothyroxine serves as the standard method of treatment for people suffering from hypothyroidism. However, a high number of people suffering from hypothyroidism treated with levothyroxine have persistently reported complaints, even though they have reached targets of therapy, which has put up the query of whether the use of levothyroxine as an alternative to thyroxine is sufficient enough to treat all the people or whether the use of alternative therapies like combination therapy using liothyronine preparations could be used as effectively [2,3]. 

## Review

Methodology

**Figure 1:**
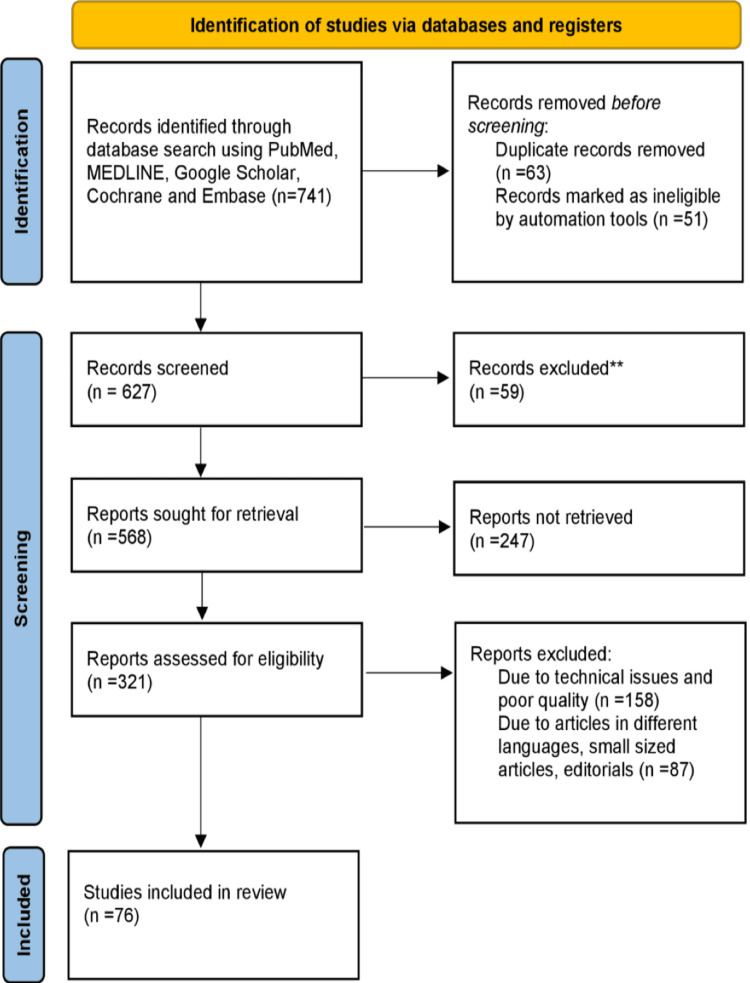
PRISMA methodology used in the study PRISMA: Preferred Reporting Items for Systematic Reviews and Meta-Analyses

Using the electronic databases PubMed, Medical Literature Analysis and Retrieval System Online (MEDLINE), Google Scholar, the Cochrane Library, and Embase, a search of the articles published or translated into English was done. The query terms were "hypothyroidism" OR "hypothyroid"; "epidemiology" OR "epidemiological data"; "prevalence" OR "incidence”; “risk factor” OR “etiology”; "treatment” OR "modalities.” The articles in this review meet the following requirements: Studies conducted exclusively on hypothyroidism, the signs and symptoms of hypothyroidism, and treatment interventions. Studies conducted in English over the last 15 years were also included. The Preferred Reporting Items for Systematic Reviews and Meta-Analyses (PRISMA) method used in research methodology is depicted in Figure 1.

Epidemiology

Risk Factors and Prevalence

The prevalence rate of primary hypothyroidism in the general population varies from 0% to 4% and 4% to 8% in the population of the USA and from 0% to 3% and 4% to 7% in the European population [4-8]. A meta-analytical study done across nine countries in Europe estimated that the prevalence of hypothyroidism that was not diagnosed, including both mild and overt cases, is estimated to be around 5%-6% [7]. The difference in the amount of iodine present in the blood has a great effect on the prevalence rate of hypothyroidism, which is found more often in people who have a higher intake of iodine than others and in people with severe iodine deficiency [9,10]. Excessive intake of iodine in iodine-sufficient patients can inhibit thyroid hormone synthesis and cause an increase in TSH. Hence, it increases the risk of hypothyroidism. Impaired thyroid function is found more often in females, in old people of the age group greater than 65 years, and also in the White population, but the data on the ethnic differences are not much [11,12]. Thyroid gland defects are commonly seen in people suffering from autoimmune diseases such as diabetes type I, celiac disease, or the presence of multiple autoimmune endocrine disorders. People suffering from Downs’ or Turners’ syndrome have greater chances of having problems with thyroid functioning. By contrast, smoking tobacco and drinking moderate amounts of alcohol may have a relationship with decreased chances of developing hypothyroidism [13,14].

Genetic Epidemiology

Heredity is the main cause of the development of hypothyroidism in individuals who have a history of hypothyroidism in their family. The chances of TSH being transferred from parents to children are 60%, while that of free T4 is 20% to 60% [15,16]. This signifies the chances of developing hypothyroidism in a healthy individual through heredity. So it is clear from these percentages that heredity is the main factor in causing hypothyroidism. The outcomes of the genome-wide association studies have only been able to explain a small amount of variability in the functions of the thyroid gland, and only three of these studies have focused on hypothyroidism specifically [17-20]. The loci in a consistent manner indicated that hypothyroidism includes genes that are related to autoimmunity and regulatory genes (thyroid-specific). A major number of them are found to be related to serum TSH levels in the normal range [17]. Mono-genetic disorders that can cause hypothyroidism since birth are not very common and have resistance to TSH (due to a mutation that inactivates the TSH receptor) and thyroid dyshormonogenesis [21].

Types and their causes

Hypothyroidism is divided into primary, secondary, tertiary, and peripheral types. Primary hypothyroidism is caused by a deficiency of T4, secondary hypothyroidism is caused by a deficiency of TSH, and peripheral hypothyroidism is caused by a deficiency of thyrotropin-releasing hormone. Secondary as well as tertiary hypothyroidism together constitute central hypothyroidism. Central and peripheral hypothyroidism (extra-thyroidal) are very rarely seen (<1%) [22].

Primary Hypothyroidism

In areas with a sufficient amount of iodine, chronic autoimmune thyroiditis, also called Hashimoto’s thyroiditis, is the main causative factor of primary hypothyroidism. High levels of anti-thyroid antibodies, which mainly involve thyroid peroxidase (TPO) as well as anti-thyroglobulin antibodies, are present in most people suffering from Hashimoto’s disease. An increase in the levels of thyroid peroxidase antibodies is found in approximately 12% of normal people [8]. In people suffering from subclinical hypothyroidism, the levels of thyroid peroxidase antibodies are very useful in determining the progression of the disease. Patients positive for TPO antibodies have a high chances of converting subclinical hypothyroidism into overt hypothyroidism [23,24]. The exact mechanisms that are involved in Hashimoto’s thyroiditis are currently unknown, but both environmental and genetic factors are a part of this mechanism. There is a need for further research into the mechanisms so that we can understand the disease in a more appropriate manner and try to prevent it. An increased genetic risk score, which was calculated using five genetic variants for thyroid peroxidase antibodies that were found by the genome-wide association studies, has shown a graded relationship between increased levels of TSH and clinical hypothyroidism [25,26]. The levels of thyroid peroxidase antibodies are lower in smokers as compared to non-smokers, and the rate of occurrence of autoimmune thyroiditis is increased after the cessation of smoking [27,28]. Other environmental factors that may cause autoimmune thyroiditis are deficiencies of vitamin D and selenium and a moderate intake of alcohol [29].

Iodine is a very important constituent of the thyroid hormone. Iodine has a paradoxical effect on thyroid function. A deficiency of iodine can cause diseases like goiter, nodules in the thyroid, and hypothyroidism. The most dangerous disorder caused by iodine deficiency is cretinism. In cretinism, there is improper physical and mental development of the baby when in the uterus as well as during the early stages of life. Iodine fortification programs are very cheap and safe public health interventions to prevent various iodine deficiency disorders [30,31]. 

Through iodine overload, iodine-containing medications (such as amiodarone) can reduce thyroid hormone production and instantly stop the production of thyroxine, also known as the Wolff-Chaikoff effect. Jod-Basedow syndrome, also known as iodine-induced hyperthyroidism, is a rare case of thyrotoxicosis due to the exogenous administration of iodine. Hypothyroidism occurs in approximately 15% of amiodarone-treated patients [32]. Lithium also contributes to hypothyroidism by impacting the production and release of thyroid hormones [33]. Within 18 months of beginning lithium therapy, 6% of participants in a sizable population-based cohort study required levothyroxine therapy [34]. For several malignancies, tyrosine kinase inhibitors are employed as targeted therapy. According to an examination of clinical records from the US Food and Drug Administration Adverse Event Reporting System, sunitinib users were more likely than sorafenib users to experience hypothyroidism [35]. Primary hypothyroidism can also be brought on by several additional medications, such as thalidomide, alfa-interferon, medications for epilepsy, and medications used as second-line treatments for multiple drug-resistant tuberculosis [36,37].

After radioiodine therapy, a hemithyroidectomy, neck radiation therapy for cancer, or cancer surgery, hypothyroidism is frequently experienced [38-40]. Even when minimal dosages of radioiodine are administered, roughly 75% of Grave's disease patients will eventually develop hypothyroidism. According to reports, 50% of patients with toxic nodular goiter who receive treatment also develop hypothyroidism and 7% of patients with isolated toxic nodules. After hemithyroidectomy, 20% of participants in a meta-analysis study experienced hypothyroidism. Infiltrative illness and transitory thyroiditis are additional main hypothyroidism causes [41,42].

Central Hypothyroidism

Central hypothyroidism is uncommon and equally prevalent in both sexes. Although it usually includes both, pituitary and hypothalamic diseases are more frequently linked to it. The biochemical characteristics of central hypothyroidism include low levels of TSH and very low levels of free T4. On rare occasions, the TSH level is slightly higher, most likely due to decreased bioactivity. Pituitary adenomas are the root cause of more than half of the cases of central hypothyroidism. Impaired functions of the pituitary gland and hypothalamus brought on by pituitary apoplexy, trauma to the head, Sheehan's syndrome, radiation, different surgical procedures, genetics, and infiltrative disease are additional causes of central hypothyroidism [43-46].

Clinical presentation and implications

Myxoedema Coma and Severe Hypothyroidism

The clinical signs and symptoms of hypothyroidism might be non-existent or life-threatening, as in the instance of myxoedema coma. The unusual illness known as myxoedema coma, which was first identified as the result of severe hypothyroidism that had gone untreated for a long time, was first documented in the late 1900s. Early detection is crucial because the disease's course is dramatic, and 40% of patients die despite receiving therapy [47]. Hypothermia, increasing lethargy, bradycardia, altered mental status, and myxoedema coma are all side effects that might finally cause multiple organ failure and death. Thyroid hormone therapy and other supportive measures must therefore be started as soon as possible [48,49]. Proper treatment and supportive care can help reduce the complications and prevent any further complications.

Signs and Symptoms

Lethargy, sensitivity to cold, an increase in weight, xeroderma, and voice distortion are some of the common symptoms of hypothyroidism in people, but the patients can also present with a variety of symptoms that differ with the gender and age of the patient and the time duration between the onset and diagnosis of the disease [50,51]. The presenting symptoms of hypothyroidism are not specific and are more commonly seen in old patients who exhibit only a few typical signs and symptoms than in younger patients [51]. Since a change in seven or more symptoms over the previous year raises the risk of hypothyroidism, a rise in symptom severity may indicate the presence of hypothyroidism [52]. In contrast, none of the 34 symptoms associated with hypothyroidism in a case-control study could be used to identify individuals who had the condition. Additionally, while 75% of people (euthyroid controls) have one or more problems related to the thyroid, only 15% of autoimmune hypothyroidism patients are found to be asymptomatic or report only one symptom linked with the condition [53].

Nearly all the major organs are affected by hypothyroidism, but the cardiovascular system has been mostly researched. Patients presented with an increase in vascular resistance, a diminishing of left ventricular functions, lower cardiac output, etc. [54]. Additionally, patients with hypothyroidism frequently have symptoms of metabolic syndrome, such as increased blood pressure (hypertension), waist circumference expansion, and elevated cholesterol levels (dyslipidemia), as well as a greater incidence of cardiovascular risk factors [55]. Additionally, the levels of homocysteine, low-density lipoprotein, and total cholesterol rise with hypothyroidism. In the course of treating thyroid cancer, patients with acute hypothyroidism exhibit a deterioration in mood and quality of life [56]. Reversible dementia is thought to be caused by hypothyroidism, although it's unclear how frequently this happens or what percentage of patients actually have reversible dementia [57]. Neurosensory, gastrointestinal (GI), and musculoskeletal signs and symptoms are additional presentations. Hypothyroidism can influence the course of other conditions due to the pleiotropic effects of thyroxine. For instance, those with hypothyroidism are more likely to experience statin intolerance than healthy controls. The signs and clinical presentation of hypothyroidism are depicted in Table 1 [58].

**Table 1:** Clinical presentation and implications of hypothyroidism

	Presentation	Signs and implications
General metabolism	The patient presents with an increase in weight, is unable to bear cold (cold intolerance), and feels exhausted most of the time (fatigue).	Increased body mass index (BMI), decreased basal metabolic rate (BMR), myxoedema, and decrease in body temperature (hypothermia)
Neuro-sensory	The patient presents with hoarseness in voice, and a decrease in sight, gustation, or hearing.	Peripheral neuritis, cochlear dysfunction, and decreased sensitivity to smell and taste
Cardiovascular system (CVS)	The patient presents with dyspnea (difficulty breathing) and fatigue from exertion.	Increase in cholesterol levels (dyslipidemia), decreased heart rate (bradycardia), increased blood pressure (hypertension), changes in the ECG
Gastrointestinal (GI)	The patient presents with difficulty in the passage of stools (constipation).	Decrease in peristalsis (movement of the esophagus), fatty liver disease
Endocrinal	The patient presents with irregular menses and infertility.	Impaired metabolism of glucose, goiter, increase in levels of the hormone prolactin, hyperplasia of the pituitary gland
Neurological and psychiatric	The patient presents with memory loss, tingling sensations, and an impaired mood.	Cognitive impairment, Woltman sign, depression, dementia, impaired coordination, carpal tunnel syndrome, myxedema coma
Musculoskeletal	The patient presents with weakness, cramps in the muscles, and joint pain.	Creatine phosphokinase elevation, fractures as a result of osteoporosis (due to too much replacement therapy)
Skin and hair	The patient presents with loss of hair (alopecia) and dryness of skin (xeroderma).	Skin becomes coarse in nature; palms turn yellowish; patchy hair loss
Hemostasis and hematological	The patient presents with fatigue and mild bleeding diathesis.	Mild anemia, decrease in the levels of protein C and protein S, increase in the red cell distribution width (RDW), increased mean platelet volume (MPV)
Electrolytes and kidney functions	The patient presents with impaired kidney functions.	Decrease in the glomerular filtration rate (GFR), deficiency of sodium (hyponatremia)

Diagnosis

According to the type of test employed and the population under study, primary hypothyroidism is characterized by TSH values higher than the standard reference range (most frequently 0.5-5 mIU/L) and free T4 levels below the reference range. The reference ranges for pregnant women are 0.1-2.5 mIU/L (first trimester), 0.2-3.0 mIU/L (second trimester), and 0.3-3.5 mIU/L (third trimester) [59].

There is fluctuation in the levels of TSH throughout the day, with the peak value achieved in the late afternoon and evening. Patients with severe hypothyroidism exhibit very high TSH secretion, which may turn erratic [60]. Seasonal changes are also found to affect TSH concentrations. Concentrations of TSH are higher in winter and spring, while they are lower in autumn and summer. These diurnal and seasonal variations don't have much effect on the severity of hypothyroidism since any effects are not much noticed and are very short-lived. Total triiodothyronine (T3), total T4, and free T3 measurements should not be done regularly. This is because changes in these values are not considered to be the positive signs of hypothyroidism and are hence neglected [61].

Reference Ranges for Thyroid Function Tests

The majority of TSH and free T4 assays used in industry are immunoassays, and their reference ranges are based on statistics to be between the 2.5th and 97.5th percentiles in a population that seems to be healthy. Studies indicating an increased risk of adverse events with variations in thyroid function even within these reference ranges prove that the reference ranges do not take symptoms or the risk of adverse events or disease into account [62-65]. Additionally, the reference ranges vary according to age, sex, and ethnicity. The TSH reference range upper limits normally rise with age, and studies from the UK and Australia found that younger people had inconsistent responses to age-specific reference ranges [66-68]. However, the adoption of age-specific reference ranges in both studies resulted in a reclassification of thyroid function from abnormal to normal, primarily in older people and pregnant women. Little is known about the effects of the treatment; hence, no strong justifications have been offered to alter the current reference ranges [69].

Treatment

The preferred treatment is swallowing a single tablet of levothyroxine on an empty stomach. The person then must not eat anything for the next hour because eating any food may hinder the absorption of the levothyroxine present in the tablet. The first sign of hypothyroidism is the presence of clinical symptoms along with laboratory evidence of overt hypothyroidism. Changing the brand of levothyroxine tablets for stable individuals is not advised. People suffering from overt hypothyroidism have to take daily medication starting at a dose of 1.5 to 1.8 mcg/kg, ideally [70-73]. Pregnant women can take a full replacement thyroxine dose of 2.0 to 2.4 mcg/kg. People suffering from coronary artery disease are advised to start taking the medications at a dosage of 12.5 to 25 mcg/day, and the dosage must be regulated based on symptoms and levels of TSH in the blood [71]. This plan of treatment is recommended for old patients, particularly those who have many comorbidities [71,72]. Patients of young age who do not have any comorbidities can often receive a sufficient or full dosage of the medication immediately, but by carefully monitoring them to prevent any case of overtreatment. Measurement of TSH levels is performed four to 12 weeks after the beginning of the therapy, then every six months until they reach a consistent phase, and subsequently annually. Adjustments in the dosage should be made according to the results of the lab tests, keeping in mind that even small dose changes might have a significant impact on serum TSH concentrations in patients with low body weight and elderly patients. Although some people reach normal TSH readings, it is unknown what clinical significance low T3 concentrations have. Levels of T3 shouldn't be routinely measured to evaluate treatment efficacy [74].

Treatment Targets

Normalization of TSH levels and relief from mental and physical symptoms are among the therapy goals, while undertreatment and overtreatment are to be avoided [71]. Nevertheless, it is believed that 35% to 60% of patients using levothyroxine (either over- or under-treated) do not achieve the desired TSH range [75,76]. Almost 6% of patients receive levothyroxine medication after five years, according to the findings of retrospective cohort research conducted in the UK, and more than 5%-10% have TSH levels above 15 mIU/L. Overtreatment should never be used, especially in postmenopausal women and elderly patients, since it might have harmful health implications such as atrial fibrillation and osteoporosis. In pregnant women, hypothyroidism should be treated with levothyroxine with a serum TSH goal of less than 2.5 mIU/L. Untreated thyroid hormone deficits (i.e., prolonged thyroid hormone shortages) might raise the risk of cardiovascular disease and leave patients with lingering signs and symptoms. Treatment targets for central hypothyroidism are different from those for primary hypothyroidism [22].

## Conclusions

Hypothyroidism is a very common endocrine disorder. It can be found in people of any age group. It is more common in women and older people. This review article describes various problems associated with hypothyroidism and summarizes epidemiological risk factors and causes that help to prevent the disease and know its root cause. The main risk factors for hypothyroidism are age and sex. Hypothyroidism is commonly seen in elderly people (>65 years) and in the female population. The main cause of hypothyroidism is heredity. Hypothyroidism has four types, of which primary hypothyroidism is the most common (99%), while the rest are very rare (<1%). Severe hypothyroidism is a fatal condition if not treated as soon as possible. Hypothyroidism affects almost all parts of the body. This article has focused on the clinical presentations and different signs and symptoms present in hypothyroid patients. The most common symptoms are weight gain, fatigue, cold intolerance, etc. Laboratory tests for the measurement of TSH and free T4 levels are the main diagnostic tests for detecting hypothyroidism. The standard treatment for hypothyroidism is levothyroxine replacement therapy. Hypothyroidism can be prevented or treated by lifestyle modifications. Therefore, eating healthy food and having the habit of exercising daily are highly recommended.
